# Risk Factors and Biomarkers for Immune-Related Adverse Events: A Practical Guide to Identifying High-Risk Patients and Rechallenging Immune Checkpoint Inhibitors

**DOI:** 10.3389/fimmu.2022.779691

**Published:** 2022-04-26

**Authors:** Adithya Chennamadhavuni, Laith Abushahin, Ning Jin, Carolyn J. Presley, Ashish Manne

**Affiliations:** ^1^University of Iowa Hospitals and Clinics, Holden Comprehensive Cancer Center, Iowa City, IA, United States; ^2^Department of Internal Medicine, Division of Medical Oncology at the Arthur G. James Cancer Hospital and Richard J. Solove Research Institute, The Ohio State University Comprehensive Cancer Center, Columbus, OH, United States

**Keywords:** immune checkpoint inhibitors, immune related adverse events, risk factors, biomarkers, predictors, colitis, pneumonitis, rechallenge

## Abstract

Immune-related adverse events (irAEs) are a range of complications associated with the use of immune-checkpoint inhibitors (ICIs). Two major classes of ICIs widely used are Cytotoxic T-Lymphocyte Antigen 4 (CTLA4) and Programmed Cell death-1 (PD-1)/Programmed death-ligand 1 (PD-L1) inhibitors. High-grade irAEs are life-threatening and often cause a severe decline in performance status in such that patients do not qualify for any further anticancer treatments. It is difficult to generalize the evidence in the current literature on risk factors or biomarkers for the entire class of ICIs as the studies so far are either disease-specific (e.g., lung cancer or melanoma) or ICI agent-specific (e.g., pembrolizumab, ipilimumab) or irAE-specific (e.g., pneumonitis or gastritis). In this review, risk factors and biomarkers to consider before initiating or monitoring ICI are listed with a practical purpose in day-to-day practice. Risk factors are grouped into demographics and social history, medical history, and medication history, tumor-specific and agent-specific risk factors. A higher risk of irAE is associated with age <60 years, high body mass index, women on CTLA4 and men on PD-1/PD-L1 agents, and chronic smokers. Patients with significant kidney (Stage IV-V), cardiac (heart failure, coronary artery disease, myocardial infarction, hypertension), and lung (asthma, pulmonary fibrosis, and chronic obstructive pulmonary disease) are at a higher risk of respective organ-specific irAEs. Pre-existing autoimmune disease and chronic use of certain drugs (proton pump inhibitors, diuretics, anti-inflammatory drugs) also increase the irAE-risk. Biomarkers are categorized into circulating blood counts, cytokines, autoantibodies, HLA genotypes, microRNA, gene expression profiling, and serum proteins. The blood counts and certain protein markers (albumin and thyroid-stimulating hormone) are readily accessible in current practice. High neutrophil-lymphocyte ratio, eosinophil/monocyte/lymphocyte counts; TSH and troponins at diagnosis and drop in the white count and lymphocyte count can predict irAE. Other biomarkers with limited evidence are cytokines, autoantibodies, HLA genotypes, microRNA, and gene expression profiling. With fast-expanding approvals for ICIs in various cancer types, knowledge on risk factors and biomarkers can help providers assess the irAE-risk of their patients. Prospective disease and agent-specific studies are needed to provide further insight on this essential aspect of ICI therapy.

## Background

Immune checkpoint inhibitor (ICI) therapy has changed the landscape of managing multiple cancer types in the last decade. They work by altering the immune regulatory pathways and thereby promoting cell-mediated destruction of the tumor cells. Several agents targeting Cytotoxic T-Lymphocyte Antigen 4 (CTLA4), programmed cell death-1 (PD-1), and Programmed death-ligand 1 (PD-L1) have been approved by the FDA (Food and Drug Administration) for multiple cancer types, and the indications for utilization is constantly expanding (1). ICIs widely used in the current clinical practice are PD-1 inhibitors (pembrolizumab, nivolumab, and cemiplimab), PD-L1 inhibitors (durvalumab, avelumab, and atezolizumab), and CTLA4 inhibitors (ipilimumab and tremelimumab). In addition, newer targets that are under investigation include LAG3 (lymphocyte activation gene-3), TIGIT (T cell immunoglobulin and ITIM domain), TIM3 (transmembrane immunoglobulin and mucin domain 3, GITR (glucocorticoid-induced tumor necrosis factor receptor [TNFR]–related), and OX40 targeting the lymphoid pathway, NKG2A, targeting the natural killer cell pathway and a bifunctional fusion protein targeting TGF-β and PD-L1 to name a few (2–10).

T cells and cancer cells have a complex regulatory mechanism. Typically, presenting major histocompatibility complex (MHC) peptide to the T cell results in cytoskeletal reorganization, downstream pathways resulting in genetic alteration, CD28 mediated co-stimulation, and an immunological synapse (11). Alternatively, several co-inhibitors such as PD-1 and CTLA – 4 on T cells prevent T cell stimulation (11). PD-L1 (B7-H1) is expressed on the surface of many cell types, including tumor cells, with PD-L2 predominantly on hematopoietic cells. During malignancy, chronic antigen presentation results in peripheral T cell exhaustion from the PD-1 and PD-L1 interaction despite the co-stimulation with CD28 (12, 13). CTLA4 has increased affinity and competes with CD28, interacting with CD80 and CD86 on antigen-presenting cells, resulting in decreased release of cytokines and cytotoxic enzymes (14). Apart from checkpoint inhibition, the addition of agents targeting tyrosine kinase inhibitors and vascular endothelial growth factors is being considered as there is evolving evidence that such combinations have more durable responses (15)

Immune-related adverse events (irAE) refer to a set of side-effects in the patients receiving ICIs similar to autoimmune responses (16). The irAE reporting is standardized with Common Terminology Criteria for Adverse Events (CTCAE) grading (17). A large meta-analysis reported all-grade incidence of irAE is about 83% with CTLA4 inhibitors, 72% with PD-1 inhibitors, and 60% PD-L1 inhibitors (18). CTLA4 inhibitors predominantly cause irAE by reinitiating exhausted T effector cells in the tumor microenvironment leading to the production of autoantigens apart from neo antigens, destroying normal tissue (19, 20). Disturbance of immune tolerance in peripheral tissues and T follicular helper cell-mediated generation of aberrant B cells mediated humoral autoimmunity are proposed mechanisms with PD1/PD-L1 inhibitors (19, 20).

The irAE profile in an individual depends on the organ that exhibits autoimmune-like activity. It may vary with the class of the ICI agent used (PD-1/PD-L1 vs. CTLA4). Compared to PD-1/PD-L1 inhibitors, CTLA4-inhibitors frequently cause colitis (irAE-GI), hypophysitis, and dermatitis (irAE-skin), and less frequently, cause pneumonitis (irAE-lung), hypothyroidism, and skeletal symptoms such as myalgias and arthralgias (21, 22). A much better understanding of the pathways involved with irAE incidence is needed to prevent them (20). It is essential to shed light on this aspect of the care given the encouraging results with ICI in the current clinical practice.

In this review, the available evidence on the risk factors contributing to irAE and biomarkers that predict the irAE occurrence was reviewed in a pattern that can be adopted in daily clinical practice. The patient’s baseline characteristics that increase the possibility of irAE before administering the first dose of ICI were classified into patient-specific risk factors and measurable entities as biomarkers. The basic history-taking sequence steps for risk factors were followed, starting with demographics (age, race, and gender), past medical history, and medication history. Few physical exam findings and social habits were covered in demographics. The tumor-specific and agent-specific risk factors were included to personalize it for a patient and the choice of the agent. The biomarkers with predictive value incorporated range from simple blood counts to complex microbiome studies. The idea is to provide helpful information for a treating physician to predict the incidence of irAEs.

The published studies that evaluated risk factors or biomarkers for irAE were historically conducted in populations that are specific to a primary tumor (example or, e.g., irAE in non-small cell lung cancer or NSCLC); ICI-agent (e.g., pembrolizumab in breast cancer, NSCLC, urothelial cancers or UC, and melanoma or MM); organ system involving irAE (e.g., pneumonitis in multiple cancers treated with PD-1 or PD-L1 or CTLA4 inhibitors); one primary and one agent (e.g., nivolumab in NSCLC); combinations (e.g., colitis in NSCLC treated with nivolumab or with PD-1 or PD-L1 or CTLA4 inhibitors in lung cancer and MM) (23–28). Therefore, it is difficult to generalize the results to an entire class of ICIs or a particular primary tumor, and the conclusions of the literature must be interpreted cautiously. It is often the combination of one or more risk factors and biomarkers that can predispose to more adverse events than one single factor/biomarker.

## Risk Factors for irAEs

**Figure 1:**
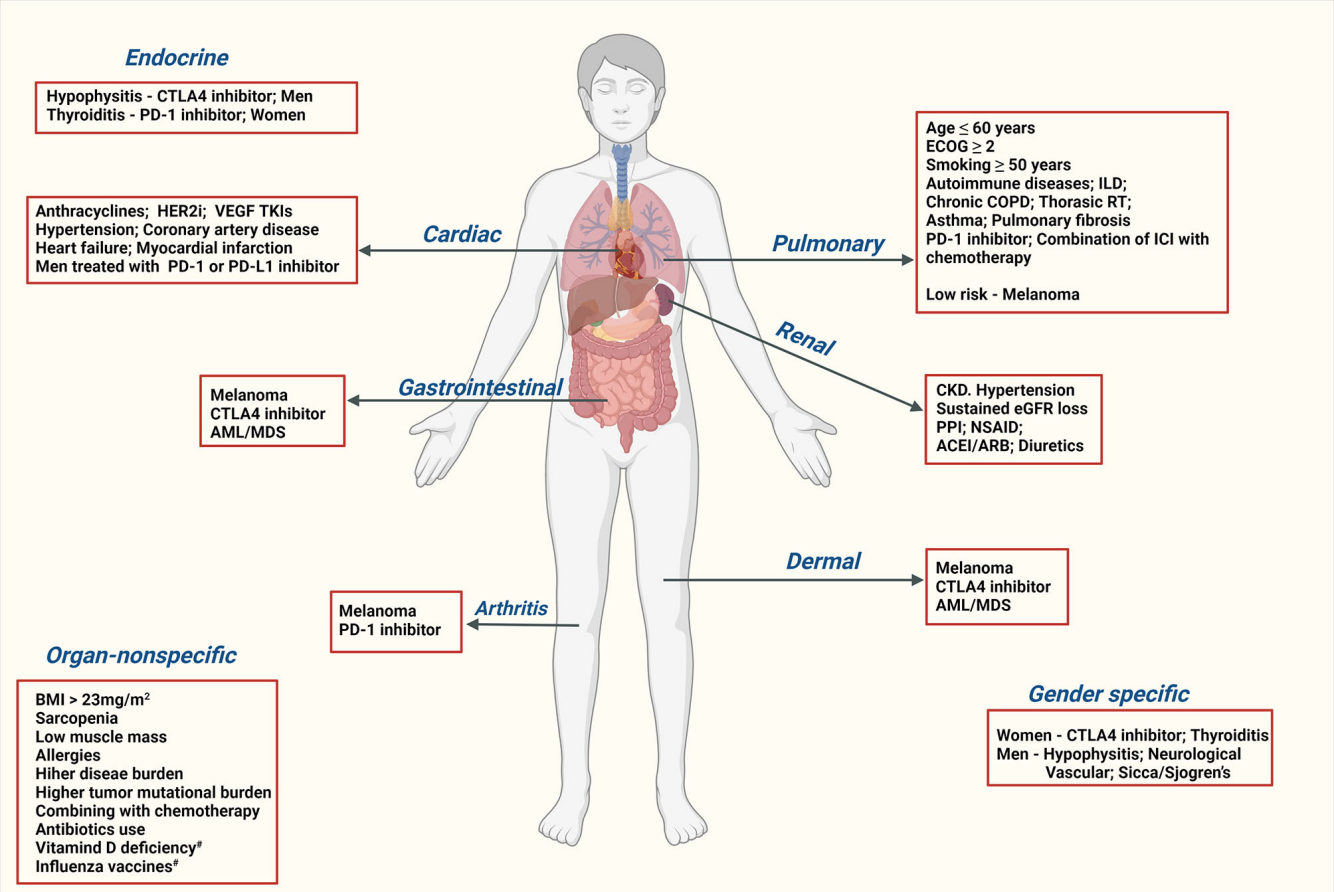
Risk factors for immune-related adverse events. CTLA4, Cytotoxic T-Lymphocyte Antigen 4; PD-1, Programmed Cell death-1; PD-L1, Programmed death-ligand 1; irAE, immune-related adverse events; HER2i, human epidermal growth factor receptor 2 inhibitors; VEGF, Vascular endothelial growth factor; TKI, Tyrosine Kinase Inhibitor; HLA, Human leukocyte antigen; AML, acute myeloid leukemia; MDS, myelodysplastic syndromes; BMI, body mass index; ECOG, Eastern Cooperative Oncology Group; ILD, interstitial lung disease; COPD, Chronic obstructive pulmonary disease; RT, radiation therapy; CKD, chronic kidney disease; AKI, acute kidney injury; PPI, Proton-pump inhibitors; NSAID, nonsteroidal anti-inflammatory drugs; ACE, angiotensin-converting enzyme inhibitors; ARB, angiotensin receptor blockers; ^#^, there is weak evidence for these risk factors.

Risk factors refer to a variable associated with an increased risk of disease or infection (29). In the context of irAEs, the risk factors were grouped into three broad categories, a) patient-specific, based on demographics, social history, past medical history, and medication history; b) tumor-specific, based on the primary tumor (organ of primary-specific or histology specific); c) agent-specific, based on the ICI used. The risk factors are summarized in **Figure 1** and **Table 1** below. The risk was measured with odds ratio or in the majority of the studies. In **Table 1**, the available OR is indicated in the parenthesis beside the risk factor. Statistically significant (p<0.05) risk factors were mentioned in **Table 1**. The possible mechanisms involved are discussed in a **Supplementary Table S1**.

**Table 1:** Risk factors and biomarkers for irAE incidence.

	Factor	High risk of irAE (odds ratio)
Patient-specific	Demographics and Social history	Age < 60 years (1.70) (30) Men in PD-1/PD-L1 (0.81) (30–32) Women in CTLA4 (1.5) (31, 33, 34) ECOG ≥ 2 (3.79) (35) Smoking of ≥ 50 pack-years (3.19) or current smoker (HR-2.26) (34–36) Higher body mass index (for ≥ 23 kg/m^2,^ OR=2.62; for ≥ 25kg/m^2^, OR=1.08) (37–39) Sarcopenia (5.34) (40) Low muscle mass (3.57) (40)
	Medical history	Autoimmune disease (2.57) (41–43). Allergies (1.48) (food, drug, or contrast) (44) Interstitial lung diseases (6.6), chronic obstructive pulmonary disease (2.79), asthma (OR = 2.82); history of thoracic radiation (3.3) (27, 45–49) Hypertension (4.3) Coronary artery disease, heart failure, myocardial infarction are more prone to cardiac-irAE (50)* Chronic Kidney disease (eGFR < 30 ml/min) (1.9) (51)
	Medication history	PPI (OR = 2.85) and NSAID (OR = 1.36) (51–56) Diuretics (OR = 4.3) and ACEI/ARD (OR = 2.9) (57) Anthracyclines, HER2 inhibitors, and VEGF TKIs (50)* Antibiotics (58) Vitamin D deficiency* (59, 60) Influenza vaccinations*^1^ (61)
Tumor-specific	Non-specific	Higher disease burden (≥ 2 metastatic sites, OR = 8.62) (24, 62) Higher tumor mutational burden^2^ (63)
	Melanoma^3^	High risk for diarrhea (1.9/1.3), rash (1.8/1.6), pruritis (2.4/1.5), colitis (4.2/not reported) (21, 34) Low risk for pneumonitis (0.4/0.3)
	Breast cancer	Higher fatal adverse events in patients with PD-1 (3.1%)^4^ (25)
	AML/MDS	High risk for dermatitis and hepatitis* (64)
Agent-specific	CTLA4 inhibitors	In combination with PD-1 (vs PD-1/PD-L1) (1.53; 1.88) ^5^ (22, 65–67) Monotherapy (vs PD-1/PD-L1) - Organ-nonspecific (2.02); Colitis (8.7); hypophysitis (6.5); and rash (2) (21)
	PD-1 inhibitors	Pneumonitis (6.4), arthralgia, vitiligo (3.5), and hypothyroidism (4.3) vs. CTLA4 (21)
	Organ-nonspecific	High risk when combined with chemotherapy (OR for grade I-V, 2.67; OR for grade III-IV, 1.83) (68)

irAE, immune-related adverse events; OR, odds ratio; all are statistically significant (p<0.05); PPI, Proton-pump inhibitors; NSAID, nonsteroidal anti-inflammatory drugs; ACE, angiotensin-converting enzyme inhibitors; ARB, angiotensin receptor blockers; HR, hazards ratio; ^1^higher rate of irAE in vaccinated compared to unvaccinated (56% vs 26%); ^2^Pearson correlation coefficient R = 0.704; P < .001; ^3^compared to Non-small cell lung cancer/Renal Cell Carcinoma; ^4^ not necessarily irAE; ^5^ OR from two studies;*these are potential risk factors.

## Patient-Specific Risk Factors for irAE

This section discusses the risk factors for irAE in the patient even before initiating ICI and further divides them into three more sets, demographics and social history, medical history, and medication history.

### Demographics and Social History

Early studies could not establish any causal relationships with demographic and social factors such as age, gender, race, smoking, or diabetes (69, 70). Even in our study on hospital-requiring irAEs in lung cancer (NSCLC and small cell) and MM patients, demographics were not significantly different in irAE groups from non-irAE groups (24). Another study out of Japan where the study population included 86 patients with NCLC, renal cell carcinoma (RCC), UC, Microsatellite instability-high (MSI-H) small bowel cancers also had similar conclusions (71).

However, other studies had different conclusions. A retrospective review of an insurance company’s database reported an increased risk of severe irAE in the younger population (72). Though statistically significant (p<0.01), the odds ratio (OR) was just 0.98 per additional year, which is not that impressive. Similarly, significant relation between age and risk of irAE-pneumonitis was reported by a pharmacovigilance study using the United States Food and Drug Administration (US FDA) Adverse Event Reporting System (FAERS) database (30). In this study, patients younger than 60 years had a higher risk of pneumonitis. A recently published review discussed the complex relationship between age and irAEs (73). The results varied with the age cut-off chosen (worse in ≥ 65 years vs < 65 years, worse in a median age group of 70 years vs. 62 years). There is also evidence for organ-specificity with age (more endocrine and gastrointestinal irAEs in younger vs dermal and rheumatological in elderly) (70, 74).

Gender and irAE risk may be dependent on the class of ICI used. Men were at high risk of pulmonary toxicity with PD-1/PD-L1 inhibitors (in multiple cancers including MM and lung cancer), while women are at high risk with CTLA-4 inhibitors (in MM) (33, 34). The kind of irAE seems to differ in men and women, too (31). Women tend to have more irAE-endocrinopathies, specifically thyroid dysfunction, and less neurological, dermal, and vascular irAEs than men. Retrospective reviews on the cardiac events in ICI therapy reported that men are at high risk of irAE-myocarditis and pericarditis along with arrhythmia, Coronary Artery Disease (CAD), and Myocardial Infarction (MI) (32, 75).

In a Korean study, patients who received pembrolizumab with a higher body mass index (BMI), ≥ 25kg/m^2^ had a significantly increased risk of irAEs (BMI: OR 1.08, 95% confidence interval [CI] 1.01-1.16) (37). This was validated by a metanalysis published in 2020 (38). High-BMI patients have a greater risk of irAE even if they have low metabolic risk (<2/3 metabolic diseases - diabetes, dyslipidemia, hypertension) (39). In a Japanese single institutional study, patients with lung cancer and a smoking history (more than 50 pack-years) and poor performance status (ECOG ≥2) had higher all-grade irAE-lung (interstitial lung disease) (35). The poor performance status alone increased the risk of severe irAE in that population. In a different study, the irAE-lung frequently occurred in men and smokers (34). In ipilumimab treated MM patients, sarcopenia and low muscle attenuation was associated with an increased risk of irAEs, according to a retrospective study by Daly et al. (40).

### Medical History

Pre-existing autoimmune disease (AD) and even a family history of it seems to increase the risk of irAE in patients treated with ICI (41–43). Contrary to most studies, a small study with 56 NSCLCs treated with PD-1 inhibitors, the incidence of irAE was not that different from the trials that excluded patients with AD (76). The risk and severity of the flare-ups may be different. For instance, inflammatory bowel disease patients are at a higher risk of flares than rheumatoid diseases (77). Studies have shown that ICIs are equally effective in AD patients but need close monitoring for flare-ups and irAEs (78, 79).

Allergies (food, drug, or contrast) by virtue of Type I hypersensitivity reactions significantly increase the risk of irAE in solid tumors (NSCLC, gastric cancer or GC, RCC, and MM), according to a retrospective study (44). There is conflicting evidence of the risk of organ-specific irAEs with organ-specific comorbidities (e.g., irAE-lung in patients with baseline lung diseases). A retrospective review out of our institution showed no significant relation between high-grade irAE incidence and any one of comorbidities (such as chronic obstructive pulmonary disease or COPD, coronary artery disease or CAD), and diabetes) (80). However, other studies identified patients with comorbidities as high risk for irAEs.

Pre-existing lung diseases such as interstitial lung diseases (ILD), pulmonary fibrosis, asthma, COPD increase the risk of irAE-pneumonitis (27, 45–49). Likewise, patients with cardiovascular risk factors such as hypertension, CAD, heart failure (HF), myocardial infarction (MI) are more prone to cardiac-irAE (50). Stage IV-V chronic kidney disease (CKD) (with estimated glomerular filtration rate or eGFR < 30 ml/min) is a reliable risk factor for acute kidney injury (AKI) secondary irAE-renal based on a multi-center study (51). The use of nephrotoxic drugs such as proton pump inhibitor (PPI), other concurrent irAE, and combination ICI therapy were other risk factors. A sustained eGFR loss (>20% decline for ≥ 90 days) was significantly associated with irAE (organ-nonspecific) than AKI (non-irAE) after initiating ICI (81). Alternatively, in a French study published this year, CKD (eGFR < 60ml/min) did not increase the risk of AKI (82). Patients with HTN and other (non-renal) irAEs are more prone to develop renal-irAE (57). In these studies, renal-irAE was confirmed by biopsy in the available tissues. On the other hand, patients with higher baseline creatinine or poor kidney function (≥ Stage III) might have more irAEs (not just renal-irAE) than patients with normal creatinine clearance (22).

### Medication History

Medications such as PPIs and nonsteroidal anti-inflammatory drugs (NSAID) are associated with kidney injury with chronic usage (51–53). Multiple studies reported worsening renal function in chronic PPI or nonsteroidal anti-inflammatory drug (NSAID) users treated with ICI (51, 54–56, 83). A case series described acute interstitial nephritis (AIN) as the common mechanism of such injury (56). Other drugs that need attention are angiotensin-converting enzyme inhibitors, angiotensin receptor blockers, diuretics, and steroids (57). Cardiotoxic drugs such as anthracyclines, human epidermal growth factor receptor 2 (HER-2) inhibitors, vascular endothelial growth factor (VEGF) and tyrosine kinase inhibitors (TKI) increases the risk of cardiac-irAE (50). Vitamin D is known to have anti-inflammatory and immune regulatory properties predominantly by dampening pro-inflammatory agent IL-17 (59, 60). Vitamin D deficiency in cancer patients receiving ICI could be associated with increased IRAE and needed to be evaluated further (84).

The debate over immune modulation associated with vaccinations (such as influenza) and its influence on ICI’s response and the risk for irAE has been ongoing for more than a decade. The majority of studies that reviewed the effect of vaccination status on increasing the irAE incidence risk found no causal relation between them (85–87). A Swiss study, on the other hand, reported higher irAE incidence post-vaccination (N=23) compared to their institutional data (historical controls) on unvaccinated patients (N=40) (61). In vaccinated population, 52% had all-grade irAE (25% ≥ grade 3) while in historical controls it was 26% (10% ≥ grade 3). A retrospective study showed a higher risk of irAEs in lung cancer patients treated with antibiotics, which was validated by other studies (58, 88).

## Tumor-Specific Risk Factors

The severity and profiles of irAEs seem to depend on histology for the primary tumor (21, 64). Head-head studies are rare to identify them. When PD-1 inhibitors are used, MM patients tend to have a) all-grade irAE-GI (colitis and diarrhea) and irAE-skin (rash and pruritis) than NSCLC patients; b) more irAE-GI (diarrhea), irAE-skin, irAE-endocrine (hypothyroidism), and irAE-musculoskeletal (arthralgia) than RCC patients; c) fewer irAE-lung (pneumonitis and dyspnea) compared to NSCLC and RCC patients d) irAE incidence is later than lung cancer patients (5.2 vs. 2.1 months) (21, 34). In myeloid malignancies such as relapsed/refractory acute myeloid leukemia (AML) and myelodysplastic syndromes (MDS) with ICI, high-grade events (≥ grade 3) usually involve the skin (15%) and liver (11%) (64).

A meta-analysis published in 2020 with 11 trials reported the fatal adverse events (FAE), not necessarily irAE, associated with pembrolizumab, were highest among breast cancer patients (3.2-1%), followed by NSCLC (2%), UC (0.8), and melanoma (0.2) (25). Combining chemotherapy with ICI worsened the risk of fatal adverse events (7% to 0.7%), but there was no significant difference between ICI and chemotherapy groups. Infectious complications were the most common cause of FAEs, followed by cardiac toxicity and pneumonitis. In another study, non-lung cancer patients such as MM and RCC were at a lower risk of hospitalization from irAEs (72).. Differences in the tumor microenvironment and microbiome composition among various cancers can explain the variations of irAE from a single ICI agent. There is also a significant disparity in utilization/pre-treatment of other treatment modalities such as chemotherapy and radiation in breast and lung cancer patients compared to patients receiving predominantly targeted therapy among MM and RCC, altering the irAE profile.

Disease burden also seems to play a role in the incidence of irAE. In our study, MM and lung cancer patients with ≥2 metastatic sites had a higher risk of severe irAE (24). In a small retrospective review on NSCLC patients (N=42), more irAE were among patients with high tumor burden (defined as the sum of the unidimensional diameters of up to five target lesions) (62). A large post-marketing study with the data from FAERS with over sixteen thousand patients with irAE reported tumor mutational burden (TMB) as an important risk factor for irAE across multiple cancer types (63). A higher median number of somatic mutations per megabase of DNA was associated with a greater risk of irAEs (Pearson correlation coefficient *R* = 0.704; P <.001)). The metastatic liver disease does not predict high-grade liver-irAE (hepatitis), but the risk is significantly higher in the patients who receive combination therapy (compared to monotherapy) (67).

## Agent-Specific Risk Factors

The incidence and profile of irAE may differ with the type of agent, PD-1, PDL1, CTLA4, or combination. Studies after studies proved the higher incidence and severity of irAE with CTLA4 inhibitors when used alone or combined with PD-1 or PD-L1 agents such as ipilimumab and nivolumab irrespective of the primary tumor treated (22, 65–67). No reliable studies proved that one class of non-CTLA4 ICI (PD-1 or PD-L1) is worse than others. Colitis, hypophysitis, and rash are frequently associated with CTLA4 inhibitors. Alternatively, pneumonitis, arthralgia, vitiligo, and hypothyroidism were common in patients treated with PD-1 inhibitors (21). A meta-analysis showed a heightened risk of irAEs in solid tumors when ICI is added to chemotherapy irrespective of the agent (and the tumor type) used (68). Likewise, there are no studies that compared irAE-profile among different (non-CTLA4) ICI agents. In summary, CTLA4 (alone or in combination) exposes patients to a higher risk of irAE, and it is not clear if one PD-1 or PD-L1 agent is better in terms of incidence or profile of irAE.

## Biomarkers for irAEs

A biomarker is defined as a characteristic measured as an indicator of normal biological processes, pathogenic processes, or responses to an exposure or intervention (89). The biomarkers discussed in this paper (as illustrated in **Table 2** below) are divided into circulating blood cell counts, cytokines, autoantibodies, serum proteins, HLA genotypes, microRNA and gene profiling, and intestinal microbiota. While the majority of the biomarker studied were irAE-organ non-specific, we indicated the biomarkers specific for irAE-GI specific and irAE-skin studies in the table.

**Table 2:** Biomarkers for predicting immune-related adverse events.

Circulating blood counts	ALC (>2.6 k/μL) (41) AEC^1^ (>240/μL; >125/μL) (90, 91) AMC (>0.29k/uL) (41) Platelet count (>145 k/μL) (41) NLR^1^ (<3; <2.3) (41, 92) dNLR (>3) (37) PLR^1^ (< 534; < 180) (26, 41). ANC (<6.5) (41) MLR (< 0.73) (41) Lower T regulatory^GI^ cells at the baseline (2.5% in irAE group versus 8.4% in non-irAE group) (93). CD8+ cells (clonal expansion > 55) (94) Early T cell receptor repertoire diversity (95) Drop-in WBC count (by 59%) and relative lymphocytic count (by 32%) from baseline ^2^ (96)
Cytokines	Lower baseline levels^2^ of TNF-α, IL-6, IL8, IP-10, CXCL9, CXCL10, CXCL11, and CXCL19. Post-treatment significant rise in the levels of IL-6, CXCL5, CXCL9, and CXCL10 levels (23, 33, 93, 97–101) Higher IL-7^2^ at the baseline and exponential rise (102) Significant rise in Granulocyte colony-stimulating factor (G-CSF) at 4 weeks compared to baseline (23) Serial Interferon-gamma (IFN-γ), decrease to <10 IU/ml 3-6 weeks after starting ICI (103) A significant rise (mean rise from 8.4mg.L to 52.7mg/L) C-reactive protein (CRP) (104)
Autoantibodies	Higher soluble CTLA-4 (>200 pg/ml) (105) Autoantibody detection - anti-GNAL, anti-ITM2B, and anti-CD74, rheumatoid factor, anti-nuclear and anti-thyroid antibody, anti-thyroid peroxidase antibody, anti-thyroglobulin antibody (106–110) Lower baseline (≤ 45μg/L) soluble major histocompatibility complex class I chain-related protein A (MICA), lower soluble CD25 (median level was 630 pg/ml in irAE group), and a significant rise in soluble CD163 (± 21.3%) (93, 101, 111) Anti-BP180 IgG (median was 6.1 U/mL) (112)
Serum proteins	Higher baseline albumin (≥3.6 g/dl) (23, 44) Significant drop in post-treatment leptin levels from baseline (23) Higher baseline thyroid-stimulating hormone levels (1.67 mIU/L) (108–110) Elevated troponin for cardiac irAE (50) lactate dehydrogenase (LDH) ≥245 U/L (88)
HLA genotypes	HLA types - HLA-DRB1*11:01^skin^ and HLA-DQB1*03:01^GI^ alleles (113) Predominance of HLA-DR4 (114) HLA-DR15, B52 and Cw12 irAE-pitutary (115). HLA-DRB1*04: 05 for irAE-arthritis (116) HLA-DPA1*02:02 and DPB1*05:01 in irAE-diabetes (117)
MicroRNA and gene expression profiling	Increased irAE risk: mapped to One variant A allele in GABRP SNP rs11743438; One variant A allele in GABRP SNP rs11743435; one variant allele A in the DSC2 SNP JHU_20.57183980; one variant allele G in the BAZ2B SNP rs56328422; one variant allele T in the SEMA5A SNP rs3026321 Post-treatment (3-week) increased expression of CD177^GI^ (12.2 fold higher in ir-AE group than non-irAE group) and CEACAM1^GI^ genes (118) Suppressed miR-146a gene (by SNP s2910164) (119) Reduced irAE risk: mapped to RGMA, ANKRD42, PACRG, GLIS3, ROBO1 genes (120).
Intestinal microbiota^GI^	Abundant Bacteroidetes phylum, specifically *Bacteroidaceae*, *Rikenellaceae*, and Barnesiellaceae (Deficient polyamine transport and vitamin B biosynthesis) (121) Abundant *Faecalibacterium* genus and other Firmicutes at baseline (93) At the phylum level, low *Bacteroidetes/Firmicutes* ratio; at the genus level, a relative abundance of *Alistipes*, low *Bacteroides*, and high *Blautia, Lachnospiraceae*, and *Faecalibacterium* (122)
Stool testing^GI^	Stool calprotectin (> 150mcg/g) and positive lactoferrin in colitis-irAE (123–127).
Cardiac workup	ECG abnormalities, and low echocardiographic global longitudinal strain (GLS) may predict cardiac-irAE (50)

^1^two values represent results of two different studies; ^2^Cut-off values are not well established; ^GI^ Specific for Gastrointesinal adverse events; ^skin^ Specific for dermal adverse events; ICI, immune check-point inhibitor; irAE, immune-related adverse events; (ALC); (AMC), absolute monocyte count; (AEC), absolute eosinophilic count; platelet count; (ABC), absolute basophil count; (NLR), neutrophil-lymphocyte ratio; (PLR), platelet lymphocyte ratio; (MLR), monocyte to lymphocyte ratio; (eGFR), Estimated glomerular filtration rate; (HER2), human epidermal growth factor receptor 2; (VEGF), Vascular endothelial growth factor; (TKI), Tyrosine Kinase Inhibitor; (HLA), Human leukocyte antigen; (AML), acute myeloid leukemia; and (MDS), myelodysplastic syndromes; SNP, single nucleotide polymorphisms.

### Circulating Blood Cell Counts

The blood counts such as absolute lymphocyte count (ALC), absolute monocyte count (AMC), absolute eosinophilic count (AEC), platelet count, absolute basophil count (ABC), neutrophil-lymphocyte ratio (NLR), platelet lymphocyte ratio (PLR), monocyte to lymphocyte ratio (MLR) have been a constant interest among clinicians and researchers as they would provide a simplest objective way to determine the probability of experiencing irAE. Multiple studies looked at various such blood counts individually or collectively. Some studies used derived NLR, which is the ratio of ANC and total white cell count – neutrophil count (37, 128). The results of various studies can be summarized as in **Table 1**. In a retrospective review of advanced NSCLC patients (on ICI), lower NLR (<3) and PLR (< 180) were frequent in the irAE group (26). Higher eosinophil and basophil counts are associated with increased endocrine and skin irAEs than other organ systems (91, 129). Higher CD4 count (median 848 vs 469, p=0.053) and among the CD4+ T cells, lower T regulatory cells (3% vs 8%, p=0.018) predicts irAE incidence (93).

After initiating ICI, a rise in WBC count (by 59%) and a drop in relative lymphocytic count (by 32%) from baseline was seen in patients having irAE-lung/GI in melanoma patients treated with nivolumab (96). One study even reported that an increase of > 3.2% of AEC after one month of initiating ICI predicts irAE (91). Similarly, expansion of CD8 T-cell clones (≥ 55) has 100% sensitivity of occurrence of irAE (G2-G3) (94). In high-risk individuals, this clonal expansion begins within two weeks of starting therapy and is diverse in CD4 and CD8 T cells with no difference in T regulatory cells compared to the low-risk population (95).

### Cytokines

As irAE is a product of over-activating the immune system, cytokines were extensively studied to predict them (23, 93, 97–100). Lower baseline levels of TNF-α, IL-6, IL8, IP-10, CXCL9, CXCL10, CXCL11, and CXCL19 are associated with high irAE risk. A significant rise in the levels of IL-6, CXCL5, CXCL9, and CXCL10 levels (from baseline) after initiating ICI is an indication of an impending irAE, and hence monitoring them might be helpful (33, 99, 101). Alternatively, higher IL-17 at the baseline with an exponential rise at six weeks is a good indicator for G3 irAE-GI (diarrhea/colitis) (102). In patients with irAE-GI, a drop in IL-17 levels correlated with the resolution of symptoms, making it a valuable indication of treatment response (28). There is some evidence of success in treating irAE (skin) with an IL-17A inhibitor (Secukinumab) (130). The irAE profile varies with the increased pro-inflammatory markers such as increased pneumonitis and colitis with IL-17, dermatitis with high IL-6 and IL-10 (97). The irAE group tends to have a uniform cytokine expression pattern, while non-irAE have a more discordant pattern. The remarkable finding in the study was a return of discordant pattern after therapy with steroids. In irAE groups, higher CXCL5 and GCSF four weeks after initiating treatment were noted in another study (23). Serial Interferon-gamma (IFN-γ) release assessed by enzyme-linked immunosorbent assay in NSCLC treated with PD-1 or PD-L1 inhibitors showed that patients with <10 IU/ml at baseline or immediately after the first dose of ICI (2-4 weeks) had more lung-irAE (pneumonitis) risk (103). C-reactive protein (CRP) is another inflammatory marker that predicts irAE (104). In 37 melanoma patients with irAE, 93% had elevated CRP (mean of 52.7mg/L) before irAE. Interestingly, 42% of them had elevated CRP before clinical symptoms.

### Autoantibodies

In an Italian study, higher pre-treatment soluble CTLA4 (>200 pg/ml) correlated with worse irAE, specifically GI-irAE in melanoma patients treated with ipilimumab (105). Autoantibodies (anti-GNAL, anti-ITM2B, and anti-CD74) detection in plasma at the baseline and on-treatment in bladder cancer and prostate cancer patients treated with ICI can also predict irAE incidence (106). Detection of auto-antibody (rheumatoid factor, anti-nuclear and anti-thyroid antibody) indicates a risk of irAE in NSCLC patients treated with ICI (107). Rheumatoid factor at the baseline specifically increases the irAE-skin. In melanoma treated with Ipilimumab, lower baseline soluble major histocompatibility complex class I chain-related protein A (MICA), lower soluble CD25, and a significant rise in soluble CD163 correlates with higher irAEs (93, 101, 111). Thyroid irAE was frequent in patients with higher baseline thyroid-stimulating hormone levels (and long duration of therapy) (108–110). The median time interval for development was three months after starting therapy. Detection of anti-thyroid peroxidase antibody at baseline and anti-thyroglobulin antibody during the therapy was significantly linked to the development of overt hypothyroidism in these patients (108–110).

### Serum Proteins

Higher baseline albumin (≥3.6 g/dl) in multiple cancers (NSCLC, MM, GC, and RCC) with PD-1 or PD-L1 inhibitors (23). Lower post-treatment leptin levels were noted in patients with irAE (23). Elevated troponin along with ECG abnormalities and low echocardiographic global longitudinal strain (GLS) may predict cardiac-irAE (50). Very few prospective trials prove using these biomarkers in clinical practice, but it is worth doing retrospective or prospective observational trials to validate them.

### HLA Genotypes

Human leukocyte antigen (HLA) refers to a set of immunogenic peptides (as receptors) on the T cells that help in distinguishing self and foreign antigens (131). In HLA typing, genes responsible for producing these antigens can be identified by DNA sequencing or polymerase chase reaction (132). HLA typing of 102 patients with NSCLC and melanoma to examine the relation between HLA allelic variations and irAE (113). The all-grade irAE risk was not significantly associated with any specific HLA gene, but when the irAE profiles were studied closely, irAE-skin (pruritus) and irAE-GI (colitis) were frequent in carriers of HLA-DRB1*11:01 and HLA-DQB1*03:01 alleles, respectively. Stamatouli et al. reviewed HLA typing of patients who developed ICI-induced autoimmune insulin-dependent diabetes and reported that HLA-DR4 is frequently (76%) associated with it (114).

### MicroRNA and Gene Expression Profiling

Preclinical data shows mice lacking MicroRNA-146a (miR-146a) had significant irAE compared to wild-type mice, along with a marked increase in neutrophils on the inflamed spleen and intestine (119). When this finding was translated in the clinical setting by performing single nucleotide polymorphism (SNP) analysis on the genomic DNA of 167 patients receiving ICI, the results were interesting. Researchers were looking for an SNP, rs2910164 (C>G), known to suppress the expression of the miR-146a gene. Patients carrying rs2910164 CC genotype (biallelic) were at a high risk of developing high-grade irAE compared with those carrying the GC or GG genotype (P = 0.004, OR = 6.78, 95% CI = 1.87–24.60) (119). Multiple other SNP associated with irAE were reported by Abdel-Wahab et al. early this year by performing next-generation sequencing on 89 melanoma patients treated with ICI (120). They identified 30 SNPs significantly associated with irAE; 12 increased the risk, while 18 reduced the risk of irAEs. GABRP, DSC2, SEM5A, OSBPL6, and AGPS were some of the genes in the former group, while RGMA, ANKRD42, PACRG, FAR2, ROBO1 were some of the genes in the latter group. There was an overlap of genes associated with autoimmune disease and irAE, such as MHC (Major histocompatibility complex), tyrosine kinase domains (TKD), and TNF alpha-induced proteins. Serial whole blood gene profiling studies in MM treated with CTLA4 inhibitor showed increased expression of CD177 and CEACAM1 genes in can predict GI-irAE incidence (118)

### Intestinal Microbiota

Microbiota refers to a collection of microbes that colonize the intestine and contribute to the host’s health. The evidence of its role in the inflammatory process has raised interest in its effect on ICI response and irAE. When the intestinal microbiota (through16S ribosomal RNA sequencing) was analyzed in 34 MM patients treated with Ipilumimab, an abundance of microbes from the Bacteroidetes phylum, specifically *Bacteroidaceae*, *Rikenellaceae*, and Barnesiellaceae is associated with reduced risk of irAE-GI (colitis) (121). Deficient genetic pathways identified (by shotgun metagenomic sequencing) with increased risk of irAE were polyamine transport and vitamin B biosynthesis. In a similar study, *Faecalibacterium* genus and other Firmicutes were abundant at baseline in patients with irAE-GI (colitis) (93). Tan et al. did serial testing of intestinal microbiota on a patient with irAE-pancreas (122). Samples were tested at the diagnosis of irAE-pancreas and after 2, 4, 6, and 13 weeks after initiating steroids. The authors got to examine the changes in the microbiota in response to treatment. At irAE incidence, at the phylum level, there was a low *Bacteroidetes/Firmicutes* ratio; at the genus level, there was a relative abundance of *Alistipes*, low *Bacteroides*, and high *Blautia, Lachnospiraceae*, and *Faecalibacterium* compared to post-treatment. They also compared the intestinal microbiota among the patients with severe or mild irAE and those with pancreatic β-cell destruction post-irAE recovery. At the genus level, the former had a relative abundance of *Alistipes*, low *Bacteroides*, and high *Lachnospiraceaewas.* Altering the microbiota with antibiotics, fecal transplant, and probiotics is still in the preclinical stage and might be in clinical practice in the coming days (133).

## Rechallenge and Prevention of irAEs

Rechallenging patients with ICIs after an irAE is another aspect of the treatment that is understudied. Various cancer societies proposed guidelines for re-initiating ICIs (134). After symptomatic recovery, it can be done while on steroid taper doses that do not affect the efficacy of the ICI (usually ≤10mg prednisone) after careful assessment of the available treatment options (135). It is tough to study the impact of rechallenging on survival, recurrence of irAE, and incidence of new irAEs as discussed in a systematic review published this year (136). It is hard to draw any concrete conclusions about the best approach with the available data as there are many variables in play such as the primary tumor, organ-involved, and ICI-class used are some of the variable factors that concrete conclusions. In a pharmacovigilance database review, when rechallenged after discontinuing ICI for irAE ≥ grade 2, only 39% experienced another ≥ grade 2 irAE, and about 70% had the same irAE at recurrence (137). The recurrence rates were also organ system-specific; it was highest in irAE-GI and least in endocrine-irAE. Time to occurrence of the first irAE seems to help predict recurrence (of irAE), as Sminonaggio et al. reported (111). In the recurrence group, the average time for the first irAE was shorter (9 weeks) compared to the non-recurrent group (15 weeks). The ideal time to rechallenge ICIs from incidence of irAE or resolution of symptoms is unclear too (136). In irAE-colitis, fecal calprotectin and lactoferrin are good biomarkers to monitor treatment response and can aid in deciding when to rechallenge the eligible patient (123–127). It is high in active irAE-colitis and drops with endoscopic and histologic remission.

Interestingly, the recurrent irAE was not as severe as the first one. Strategies advised for rechallenging and prevention are included in **Table 3**. Currently, there are no successful strategies for preventing irAE. American Gastroenterology Association published guidelines for managing irAe-colitis and irAE-hepatitis in March of 2021 (140). Budesonide is not beneficial for irAE-colitis prophylaxis. Similarly, there is no good evidence to use prophylactic steroids or any anti-inflammatory drugs in patients without any history of irAE. All the ICIs in the current practice have fixed doses, and there is no scope for modifying the dose as we might do while using chemotherapy.

**Table 3:** Rechallenging immune-checkpoint inhibitors and prevention of immune-related adverse events.

Rechallenge	Class switch: PD-1/PD-L1 to CTLA4, vice versa (43, 138)
	Resume same agent: After complete recovery from irAE (34, 135, 139)
	Drop CTLA4 agent: In patients who had irAE with CTLA4 and PD-1 combination (140)
Prevention	Testing inflammatory markers such as lactoferrin and calprotectin in patients with ≥ grade 2 diarrhea
	For the patient with grade 1 hepatitis, liver chemistries must be repeated 1-2 times/week
	For patients with G2 hepatitis, therapy must be held until resolution to G1

## Discussion

In the current clinical practice, ICI use is growing exponentially. Limited prospective studies explored the factors contributing to the irAE. Retrospective studies were done in very specific populations, and it is difficult to draw solid conclusions and apply them to all cancers or agents or irAE types. An insight into the risk factors and biomarkers with significant predictive value for irAE incidence will enable the treating clinician to take the necessary steps to protect the patients from irAE and continue benefiting with ICI. In real-world practice, knowledge on organ-specific risk, gender-specific, comorbidity or medication-specific, primary tumor-specific, and ICI-agent-specific risk factors and biomarkers will allow physicians to personalize the necessary monitoring strategies to prevent irAE and restart ICIs post-irAE.

Patients must be trained to identify the irAE and alert the treating team early. High-risk individuals (chronic smokers, poor performance status, high BMI, and sarcopenia) must be monitored closely for irAE. Kidney injury (AKI) associated with ICI is frequent in patients with CKD. It is advisable to hold PPIs and NSAIDs or switch them to alternatives before initiating ICIs. The irAE- pneumonitis is frequent in patients with pulmonary fibrosis, asthma, and COPD. Patients with significant cardiac disease history (MI, HF) and uncontrolled hypertension may have more cardiac adverse events such as myocarditis or pericarditis. Patients receiving HER2 inhibitors, VEGF TKIs, and anthracyclines in prior lines of therapy should be monitored for cardiac irAEs.

Circulating blood counts and TSH/T4 are the only biomarkers accessible in the current clinical practice and must be used when possible. Absolute counts such as ALC, AEC, platelet count, or ratios such as NLR and PLR can predict irAE incidence. Following WBC and ALC might help monitor patients on ICI for irAEs. Prospective studies are needed to shed further light on other biomarkers such as cytokines, HLA genes, microRNAs, gene expression profiling, and intestinal microbiome need more studies before they can be adopted in daily practice.

Patients having an excellent response to ICI can be rechallenged based on the initial irAE grade and tolerability with careful assessment of risks and benefits. Resumption of the same agent after full recovery is popular, but class switching can be tried. Dropping CTLA4 inhibitors is also advised in patients with irAE on combination therapy. Close monitoring of liver chemistries (every week) for grade 1 hepatitis on ICI therapy and holding the therapy for grade 2 hepatitis is advised. Checking inflammatory markers (lactoferrin and calprotectin) for patients with diarrhea might help in the early diagnosis of colitis.

The majority of ongoing trials with ICIs are doing correlative studies by collecting patients’ blood, tumor, and microbiome. The focus of research is shifting towards incorporating genomic data (mutations or methylation) and tumor microenvironment (T-cells) from accessible sources such as blood, stool, and urine to study the factors affecting toxicity and efficacy of ICIs. Acknowledging the need to do more prospective studies in this unexplored facet of ICI therapy, these risk factors and biomarkers list can be a good starting point in clinical practice.

## Author Contributions

AC and AM contributed to the conception and design of the study. AC and AM organized the database, selected relevant articles, and wrote the first draft of the manuscript. Multiple revisions and additions to the final manuscript are contributed by LA, NJ, and CP. All authors contributed to manuscript revision, read, and approved the submitted version.

## Conflict of Interest

The authors declare that the research was conducted in the absence of any commercial or financial relationships that could be construed as a potential conflict of interest.

## Publisher’s Note

All claims expressed in this article are solely those of the authors and do not necessarily represent those of their affiliated organizations, or those of the publisher, the editors and the reviewers. Any product that may be evaluated in this article, or claim that may be made by its manufacturer, is not guaranteed or endorsed by the publisher.
