# Supplementary material for: Risk Factors and Biomarkers for Immune-Related Adverse Events: A Practical Guide to Identifying High-Risk Patients and Rechallenging Immune Checkpoint Inhibitors
Source: Front Immunol. 2022 Apr 26;13:779691. doi: 10.3389/fimmu.2022.779691 (PMC9086893; doi:10.3389/fimmu.2022.779691)
Supplement: Supplementary file 1 [file DataSheet_1.pdf]

Supplementary Table S1: Potential mechanisms that increase the risk of immune-related adverse event incidence

Risk factor or biomarker	Possible mechanism for increasing the risk of irAE
Age	Age related changes to immune system (immunosensence) [1]
Gender	Immunomodulation by hormones - Generally, estrogens are considered enhancers of immune cells activity while progesterone and androgens are immunosuppressants [2]
Higher body mass index	Increased leptin in obesity is pro-inflammatory [3]
Sarcopenia and low muscle mass	Altered metabolism and clearance of anticancer drugs, and pro inflammatory state [4]
Allergy	Activation of allergen-specific CD4+ T cells [5]
Autoimmune disease or autoimmune antibody detection	Common human leukocyte antigen (HLA) loci involved [6]
Comorbidities	Exacerbation of preexisting conditions and vasculitis [7].
Medications	Changes in microbiome (antibiotics) or may induce loss of tolerance of memory T cells that have previously been primed to a drug or other hapten (ACE/ARB, NSAID or PPI) [8]
Vitamin D	Mediates downregulation of IL-17A and upregulation of IL-10 resulting in IFN-β mediated effect on CD4+ T [9, 10]
Influenza vaccine	Viral-based vaccination mediated infiltration of central memory T cells into the tissues, leading to irAE [11]
Higher tumor mutational burden	Higher neoantigenic load leading to more immunogenicity [12]
Immune system related biomarkers - Circulating blood cells (white	Dynamic alteration in tumor microenvironment, macrophage activation and fluctuations in signaling cytokines along with pro

blood cells – all kinds and platelets) and cytokines	inflammatory conditions are responsible for increased irAEs [6, 13-21]
Serum proteins	Nutritional status can influence the degree of systemic immune activation and higher irAEs [18]
Specific gene expression and micro-RNA profiling	Increased inflammatory response and predisposition to autoimmune disease [22-24]
Intestinal microbiota	Variable levels of Tregs levels in gut influenced by flora can lead to significant irAEs [17, 25, 26]

irAE- immune related-adverse events; PPI - Proton-pump inhibitors; NSAID - nonsteroidal anti-inflammatory drugs; ACE angiotensin-converting enzyme inhibitors, ARB angiotensin receptor blockers.

1. Wong, S.K., C.A. Nebhan, and D.B. Johnson, *Impact of Patient Age on Clinical Efficacy and Toxicity of Checkpoint Inhibitor Therapy.* Frontiers in Immunology, 2021. **12**.
2. Triggianese, P., et al., *Immune checkpoint inhibitors-induced autoimmunity: The impact of gender.* Autoimmunity Reviews, 2020. **19**(8): p. 102590.
3. Guzman-Prado, Y., J. Ben Shimol, and O. Samson, *Body mass index and immune-related adverse events in patients on immune checkpoint inhibitor therapies: a systematic review and meta-analysis.* Cancer Immunology, Immunotherapy, 2021. **70**(1): p. 89-100.
4. Daly, L.E., et al., *The impact of body composition parameters on ipilimumab toxicity and survival in patients with metastatic melanoma.* British Journal of Cancer, 2017. **116**(3): p. 310-317.
5. Shimozaki, K., et al., *Analysis of risk factors for immune-related adverse events in various solid tumors using real-world data.* Future Oncology, 2021.
6. Michailidou, D., et al., *Association of blood biomarkers and autoimmunity with immune related adverse events in patients with cancer treated with immune checkpoint inhibitors.* Scientific Reports, 2021. **11**(1).
7. Pirozzi, F., et al., *Cardiovascular Toxicity of Immune Checkpoint Inhibitors: Clinical Risk Factors.* Current Oncology Reports, 2021. **23**(2).
8. Franzin, R., et al., *The Use of Immune Checkpoint Inhibitors in Oncology and the Occurrence of AKI: Where Do We Stand?* Frontiers in Immunology, 2020. **11**.
9. Dankers, W., et al., *Human Memory Th17 Cell Populations Change Into Anti-inflammatory Cells With Regulatory Capacity Upon Exposure to Active Vitamin D.* Front Immunol, 2019. **10**: p. 1504.
10. Joshi, S., et al., *1,25-dihydroxyvitamin D(3) ameliorates Th17 autoimmunity via transcriptional modulation of interleukin-17A.* Mol Cell Biol, 2011. **31**(17): p. 3653-69.
11. Läubli, H., et al., *Influenza vaccination of cancer patients during PD-1 blockade induces serological protection but may raise the risk for immune-related adverse events.* Journal for ImmunoTherapy of Cancer, 2018. **6**(1).
12. Y, S., et al., *The association between tumor burden and severe immune-related adverse events in non-small cell lung cancer patients responding to immune-checkpoint inhibitor treatment.* Lung cancer (Amsterdam, Netherlands), 2019. **130**.

13. Tyan, K., et al., *Cytokine changes during immune-related adverse events and corticosteroid treatment in melanoma patients receiving immune checkpoint inhibitors.* Cancer Immunology, Immunotherapy, 2021.
14. Kang, D.H., et al., *Baseline Serum Interleukin-6 Levels Predict the Response of Patients with Advanced Non-small Cell Lung Cancer to PD-1/PD-L1 Inhibitors.* Immune network, 2020. **20**(3): p. e27-e27.
15. Khan, S., et al., *Immune dysregulation in cancer patients developing immune-related adverse events.* British Journal of Cancer, 2019. **120**(1): p. 63-68.
16. Lim, S.Y., et al., *Circulating Cytokines Predict Immune-Related Toxicity in Melanoma Patients Receiving Anti-PD-1–Based Immunotherapy.* Clinical Cancer Research, 2019. **25**(5): p. 1557-1563.
17. Chaput, N., et al., *Baseline gut microbiota predicts clinical response and colitis in metastatic melanoma patients treated with ipilimumab.* Ann Oncol, 2017. **28**(6): p. 1368-1379.
18. Oyanagi, J., et al., *Predictive value of serum protein levels in patients with advanced non-small cell lung cancer treated with nivolumab.* Lung Cancer, 2019. **132**: p. 107-113.
19. Valpione, S., et al., *Sex and interleukin-6 are prognostic factors for autoimmune toxicity following treatment with anti-CTLA4 blockade.* Journal of Translational Medicine, 2018. **16**(1).
20. Fujimura, T., et al., *Serum levels of soluble CD163 and CXCL5 may be predictive markers for immune-related adverse events in patients with advanced melanoma treated with nivolumab: a pilot study.* Oncotarget, 2018. **9**(21): p. 15542-15551.
21. Eun, Y., et al., *Risk factors for immune-related adverse events associated with anti-PD-1 pembrolizumab.* Scientific Reports, 2019. **9**(1).
22. Abdel-Wahab, N., et al., *Genetic determinants of immune-related adverse events in patients with melanoma receiving immune checkpoint inhibitors.* Cancer Immunology, Immunotherapy, 2021.
23. Shahabi, V., et al., *Gene expression profiling of whole blood in ipilimumab-treated patients for identification of potential biomarkers of immune-related gastrointestinal adverse events.* Journal of Translational Medicine, 2013. **11**(1): p. 75.
24. Marschner, D., et al., *MicroRNA-146a regulates immune-related adverse events caused by immune checkpoint inhibitors.* JCI Insight, 2020. **5**(6).
25. Dubin, K., et al., *Intestinal microbiome analyses identify melanoma patients at risk for checkpoint-blockade-induced colitis.* Nat Commun, 2016. **7**(1): p. 10391.
26. Tan, B., et al., *Clinical-radiological characteristics and intestinal microbiota in patients with pancreatic immune-related adverse events.* Thorac Cancer, 2021. **12**(12): p. 1814-1823.
